# Variation in Occurrence and Aflatoxigenicity of *Aspergillus flavus* from Two Climatically Varied Regions in Kenya

**DOI:** 10.3390/toxins12010034

**Published:** 2020-01-06

**Authors:** Ethel Monda, Joel Masanga, Amos Alakonya

**Affiliations:** 1 Department of Biochemistry, Biotechnology and Microbiology, Kenyatta University, Thika Road, Nairobi P.O. Box 43844-00100, Kenya; ethelmonda@ku.ac.ke (E.M.); masanaga.joel@gmail.com (J.M.); 2 Seed Health Unit, Genetic Resources Program, International Maize and Wheat Improvement Center (CIMMYT), Carretera Mexico-Veracruz Km. 45 El Batan, Texcoco, Mexico C.P. 56237, Mexico

**Keywords:** aflatoxins, agro-ecology, *Aspergillus flavus*, biological control, climate change, cropping systems, microbial diversity

## Abstract

Aflatoxins are carcinogenic chemical metabolites produced by *Aspergillus* spp. of the section *Flavi.* In Kenya, *Aspergillus flavus* is the most prevalent and has been associated with several acute and chronic aflatoxin outbreaks in the past. In this study, we evaluated the occurrence of *A. flavus* in soils from two agro-ecological regions with contrasting climatic conditions, aflatoxin contamination histories and cropping systems. *Aspergillus* spp. were first isolated from soils before the identification and determination of their aflatoxigenicity. Further, we determined the occurrence of *Pseudomonas* and *Bacillus* spp. in soils from the two regions. These bacterial species have long been associated with biological control of several plant pathogens including *Aspergillus* spp. Our results show that *A. flavus* occurred widely and produced comparatively higher total aflatoxin levels in all (100%) study sites from the eastern to the western regions of Kenya. For the western region, *A. flavus* was detected in 4 locations (66.7%) that were previously under maize cultivation with the isolates showing low aflatoxigenicity. *A. flavus* was not isolated from soils under sugarcane cultivation. Distribution of the two bacterial species varied across the regions but we detected a weak relationship between occurrence of bacterial species and *A. flavus*. We discuss these findings in the context of the influence of climate, microbial profiles, cropping systems and applicability in the deployment of biological control remedies against aflatoxin contamination.

## 1. Introduction

Contamination of food and feed by mycotoxins is a worldwide problem that negatively impacts human and animal health [1,2,3,4,5,6,7]. Further, high contamination levels in agricultural commodities hinder trading at the international level [8]. Aflatoxins are a group of secondary fungal metabolites primarily produced by fungi belonging to *Aspergillus* section *Flavi* [9,10]. When ingested through consumption of contaminated food and feed, these metabolites pose serious health risks to both humans and animals [11]. The health risks associated with aflatoxin ingestion can be either acute or chronic. Because of these health risks, various countries and health organizations have set maximum exposure limits [12].

Several members of section *Flavi* produce aflatoxins but *A. flavus* is commonly associated with aflatoxin contamination of feed and food worldwide [9,10,11,13,14,15]. *A. flavus* is predominantly a saprophytic fungus residing in the soil and colonizes various environments with rich sources of carbon and nitrogen [16,17]. Various aflatoxin contamination control strategies have been proposed [18,19]; key among them being pre-harvest strategies that include the application of atoxigenic *A. flavus* strains at the pre-silking stage and good agricultural practices comprising early harvesting and proper drying of the harvested grains to moisture levels below 13% [17,20,21]. However, one major factor that could affect the success of such interventions is knowledge of the level of fungal inoculum in the soil as well as the toxigenicity of the existing isolates. Diverse populations of *A. flavus*, with varying degrees of aflatoxigenicity have been reported across tropical and temperate regions and in fields with different crops [22,23]. Furthermore, high and low producers of aflatoxins have been isolated across various countries in Africa including Kenya and Nigeria [2,24,25].

The use of other microbes in pre-harvest aflatoxin control has also been widely demonstrated and is closely associated with competitive exclusion of toxigenic strains [26,27,28]. Application of atoxigenic *A. flavus* strains was shown to reduce aflatoxin contamination by toxigenic *A. flavus* in maize, cottonseed and groundnuts [13,17]. Several mechanisms have been implicated in the efficacy of bio-control agents with either competitive exclusion of toxigenic *A. flavus* by atoxigenic ones or biosynthesis of antifungal compounds, that inhibit or completely arrest growth of mycotoxin-producing fungi [13,29,30]. The ability of fungi to colonize crops, survive and produce toxins is also affected by a range of environmental conditions that include temperature, rainfall and relative humidity [29,31]. It has further been shown that abiotic stresses such as drought conditions and higher temperatures can result in an increase in production of aflatoxins [32]. Although, the distribution, population structure and aflatoxin production profiles of *Aspergillus* species have been widely studied in Kenya [23,33,34,35,36], information on the role played by different environmental factors across agro-ecological zones is limited. Agro-ecological Zoning (AEZ) refers to the division of an area of land into smaller units, which have similar characteristics related to land suitability, potential production and environmental impact. In addition, knowledge of how microorganisms especially bacteria influence the toxigenecity of *A. flavus* is lacking. In the context of this work we compare the eastern region (semiarid) and the western region (sub-humid-semi humid) [34]. The objective of this study, therefore, was to determine the microbial profiles of soils from fields with different cropping patterns and under varied environmental conditions in western and eastern regions of Kenya with the aim of associating such factors with potential for aflatoxin contamination in the two regions.

## 2. Results

### 2.1. Distribution of Aspergillus flavus across Eastern and Western Regions in Kenya

**Figure 1:**
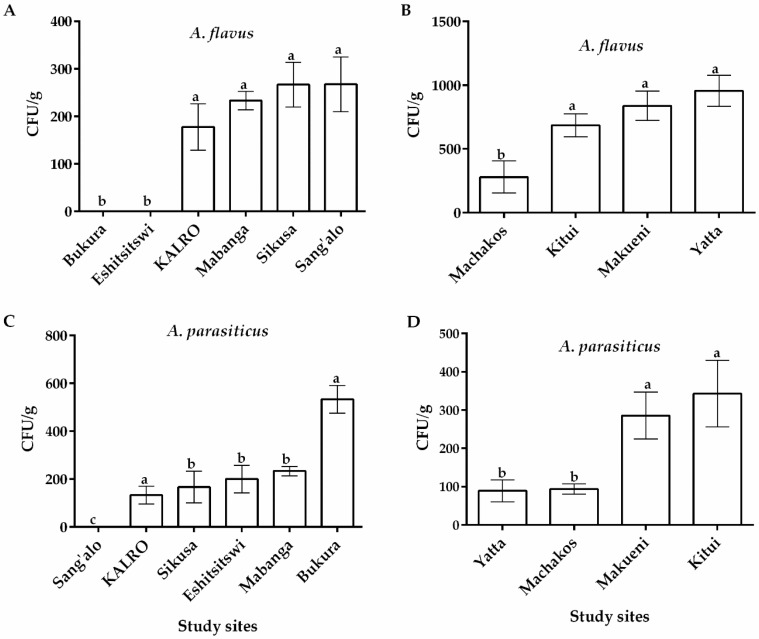
Occurrence of *Aspergillus flavus* and *A. parasiticus* (CFU/g) isolated from soils sampled from maize farms in eastern and western regions of Kenya. Quantity of *A. flavus* in western (**A**) and eastern (**B**) regions Quantity of *A. parasiticus* in western (**C**) and eastern (**D**) regions. Vertical bars represent standard deviations of the means. Means from same fungal species and region that are followed by the same letter are not significantly different (*p* > 0.05).

Differences in colony color and conidial morphology on modified Rose Bengal agar (MRBA) allowed correct identification of the fungi and differentiation of *Aspergillus* spp. from the rest. Colonies with a yellowish green color on potato dextrose agar (PDA) and an intense yellow-orange reverse color on *Aspergillus-flavus-parasiticus* agar (AFPA) medium were selected as *Aspergillus* section *Flavi.* Distribution of fungi across the two regions in Kenya was varied and is summarized in Figure 1. Particularly, *Aspergillus flavus* was recovered from soils from all sample locations in the eastern region and 4 out of the 6 locations in the western region of Kenya. All these fields had previously been under maize cultivation (Figure 1). Among the study sites in the eastern region of Kenya, Yatta recorded the highest occurrence of *A. flavus* with an average of 955.33 ± 22.33 CFU/g of soil followed by Makueni (838.057 ± 115.36), Kitui (685.671 ± 290.86) and Machakos (280.94 ± 27.14) (Figure 1B). Among the study sites with *A. flavus* occurrence in the western region, Sang’alo, Sikusa and KALRO recorded the joint highest average colony forming units per gram (CFU/g) of soil (267.67 ± 57.73) while Mabanga had the lowest (233.35 ± 19.26) (Figure 1A). There were significant differences (*p* ≤ 0.05) in average *A. flavus* CFU/g of soil between eastern and western (Table 1). A high number of *Aspergillus parasiticus* was also isolated from all study sites in eastern and 5 out of the 6 study sites in the western region of Kenya (Figure 1C,D). However, there were no significant differences (*p* > 0.05) in average *A. parasiticus* CFU/g of soil between eastern and western regions (Table 1). Similarly, no significant differences were recorded in *Trichoderma viride* between western and eastern regions (Table 1). In addition to *A. flavus*, *A. parasiticus* and *Trichoderma viride*, other fungal genera including *Penicillium* spp. and *Fusarium* spp. were also isolated from soil samples in both regions (data not shown). 

**Figure 2:**
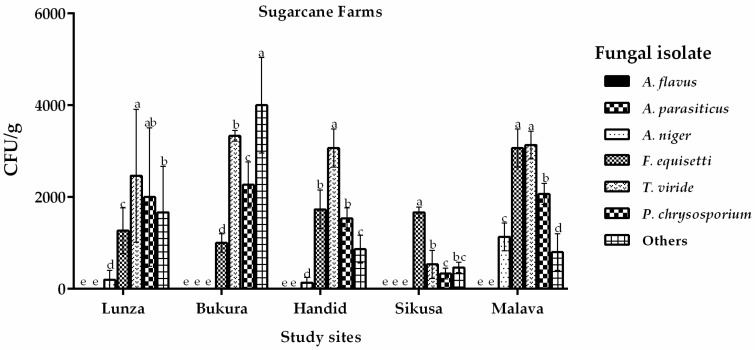
Occurrence of fungi (CFU/g) isolated from soils sampled from sugarcane farms in the western region of Kenya. Vertical bars represent standard deviations of the means. Means that are followed by the same letter and are from same site are not significantly different at *p* > 0.05.

On the other hand, analysis of soils from farms under sugarcane cultivation did not recover any *Aspergillus* section *Flavi* isolates (0 CFU/g) (Figure 2). Nevertheless, other fungi like *Aspergillus niger*, *Fusarium equisetti*, *Trichoderme viride* and *Phanerochaete chrysosporium* were recovered *(*Figure 2). Clearly, the cropping pattern had an influence on occurrence of *A. flavus* in the western region (Figure 2).

### 2.2. Molecular Analysis of Aspergillus flavus 

**Figure 3:**
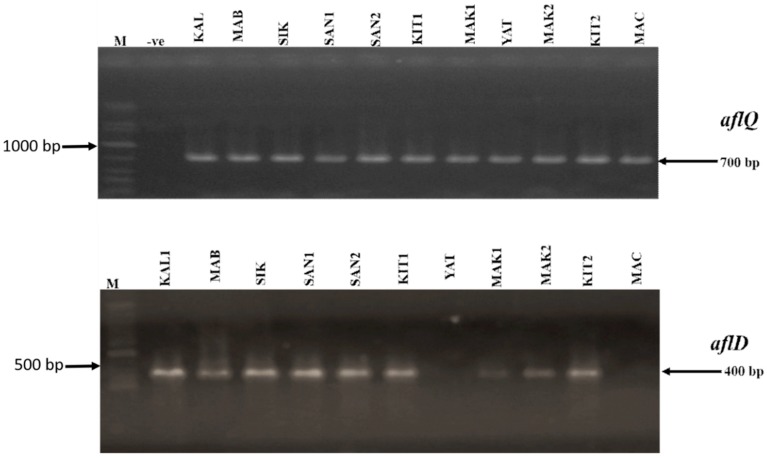
Profiles of the polymerase chain reaction (PCR) amplification of *aflQ* and *aflD* genes in *A. flavus* isolates from eastern and western regions of Kenya. M-1Kb DNA ladder (Bioline), -ve-negative control with water as template, KAL1-KALRO; MAB2-Mabanga; SIK-Sikusa; SAN1 and SAN2- Sang’alo; KIT1-Kitui; MAK1-Makueni; YAT-Yatta; MAK2-Makueni 2; KIT2-Kitui 2; MAC-Machakos.

From a total of 26 *A. flavus* isolates from the western region (KARLO n = 5; Mabanga n = 6 Sikusa n = 7 and Sang’alo n = 8) and 51 isolates from the eastern region (Yatta n = 10; Kitui n = 12; Makueni n = 19 and Machakos n = 11), we performed a neutral red desiccated coconut agar (NRDCA) pre-screening assay as described by Atanda et al. [37]. From the assay we determined that all isolates from KARLO, Mabanga and Sang’alo showed low florescence while all from Sikusa showed mid florescence. We therefore selected one isolate as a representative for each of the areas sampled in the western region. For the eastern region, due to a high number of isolates recovered and also based on the differences in fluorescence profile of isolates from every sampled location as—All isolates from Yatta showed low fluorescence, while in Kitui all isolates showed high fluorescence, in Makueni 5 isolates showed mid fluorescence while 14 isolates showed high fluorescence and all isolates in Machakos showed medium fluorescence. We therefore selected representative isolates as follows—Kitui (2 isolates), Yatta (1 isolate), Machakos (1 isolate) and Makueni (1 isolate). Based on this pre-screening we selected a total of 11 isolates for polymerase chain reaction (PCR) analysis. Polymerase chain reaction analysis of the cultured *A. flavus* revealed 700 bp and 400 bp fragments of the *aflQ* and *aflD* genes. All the isolates from both regions were positive for *aflQ* while 9 out of the 11 cultures screened across both regions were positive for *aflD* gene. Two (2) isolates from the eastern region (one each from Yatta and Machakos) did not show amplification for *alfD* (Figure 3). 

### 2.3. Occurrence of Bacteria across Eastern and Western Regions of Kenya

**Figure 4:**
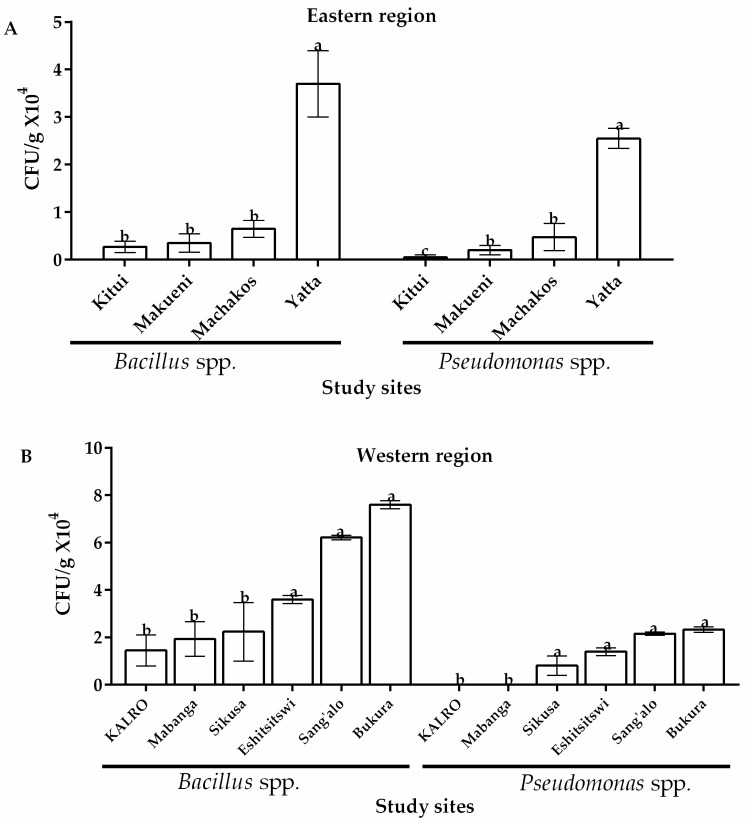
Occurrence of *Bacillus* and *Pseudomonas* spp. (CFU/g) isolated from soils in (**A**) eastern and (**B**) western regions of Kenya. Vertical bars represent standard deviations of the mean. Means followed by the same letter and of same bacterial species from same region are not significantly different at *p* > 0.05.

Two bacterial species of potential importance in biological control of *A. flavus* were isolated from both regions and their occurrence across study sites is as outlined in Figure 4. *Bacillus* and *Pseudomonas* spp. were identified and confirmed. With regard to their occurrence, *Bacillus* spp. were isolated from all soil samples from the study sites in eastern and western regions of Kenya although the latter region showed comparatively higher occurrence of this species (Figure 4). Among eastern locations, Yatta recorded the highest occurrence of *Bacillus* spp. with 4.3 × 10^4^ CFU/g of soil while Kitui had the lowest (0.27 × 10^4^ CFU/g of soil) (Figure 4A). In the western region, Bukura recorded the highest occurrence of *Bacillus* spp. (7.6 × 10^4^ CFU/g of soil). Significantly lower levels (*p* ≤ 0.05) were obtained across the other sites with KALRO recording the lowest CFU/g (1.44 × 10^4^) (Figure 4B). Relatively lower occurrences of *Pseudomonas* spp. were recorded in the two AEZs with 75% and 66.7% of sites showing occurrence in western and eastern regions of Kenya, respectively. Of these sites, Yatta recorded the highest occurrence of *Pseudomonas* (an average 2.55 × 10^4^ CFU/g of soil) in the eastern region while Sang’alo had the highest occurrence (2.3 × 10^4^ CFU/g of soil) in the western region (Figure 4). A t-test, however, revealed that the occurrence of both *Pseudomonas* spp. and *Bacillus* spp. across the 2 regions was not significantly different (*p* > 0.05) (Table 1).

### 2.4. Phylogenetic Analysis of Bacterial Isolates

**Figure 5:**
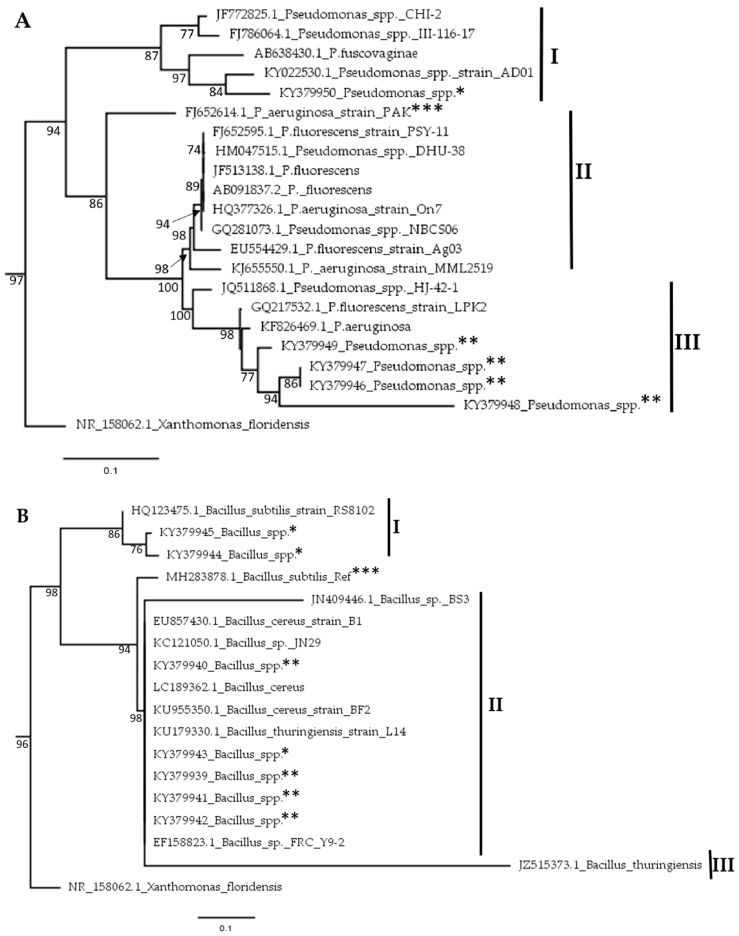
Phylogenetic analysis of; (**A**) *Pseudomonas* spp. and (**B**) *Bacillus* spp. isolates sampled from soils in eastern and western regions of Kenya. Phylogenetic trees were generated using the Maximum Likelihood method using MEGA 7.0 software [38]. Isolates from this study are marked with ** for the western region while those from the eastern region are marked with *, while reference sequences for both genera under study are marked with ***.

A total of five (n = 5) *Pseudomonas* and seven (n = 7) *Bacillus* isolates were identified in the soils across the two regions. Using sequences from our Sanger sequencing and the existing ones at NCBI, evolutionary relationships among these isolates were evaluated through a phylogenetic analysis. With regards to *Pseudomonas*, the nodes on the phylogenetic tree were divided into 3 sub families designated I, II and III (Figure 5A). According to the Maximum likelihood (ML) tree, our *Pseudomonas* isolates clustered in sub families I and III. One isolate was found in sub family I with a 100% identity to KY022530.1; a *Pseudomonas* spp. isolated from Nigeria. The rest of our *Pseudomonas* isolates clustered closely to KF826469.1; a *Pseudomonas aeruginosa* isolate from India. Similarly, the *Bacillus* isolates also clustered into 3 subgroups (Figure 5B). Five of these from the western region clustered in subfamily II. In this group, the isolates clustered closely with LC189362.1, a *Bacillus cereus* isolate from Indonesia. The rest of our *Bacillus* isolates could be found in subfamily I in a close relationship with *Bacillus subtillis*, isolated from cotton in China (Figure 5B).

### 2.5. Relationship between Occurrences of A. flavus and Bacteria

**Figure 6:**
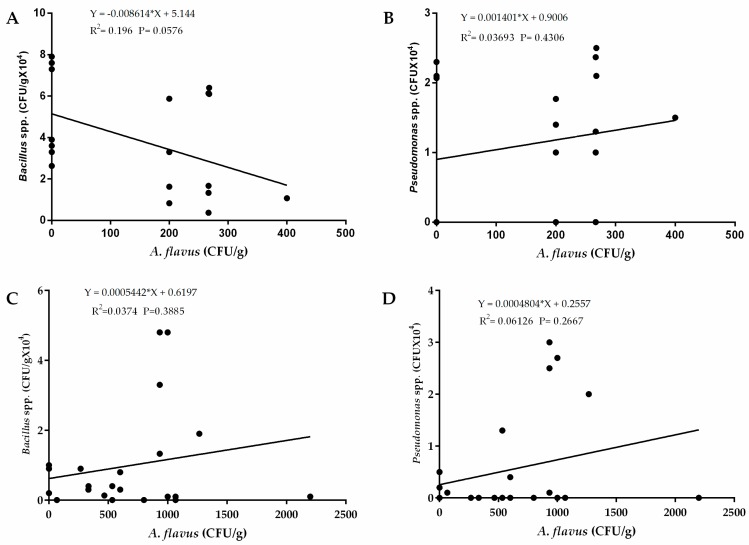
Regression analysis between occurrence of *A. flavus* and *Pseudomonas* spp. and *Bacillus* spp. in soils sampled from western and eastern regions of Kenya. (**A**) Regression analysis between *A. flavus* and *Bacillus* spp.; (**B**) and *Pseudomonas* spp. in the western region of Kenya. (**C**) Regression analysis between *A. flavus* and *Bacillus* spp.; (**D**) and *Pseudomonas* spp. in the eastern region of Kenya. The vertical axis represents average occurrence (CFU/g) of bacteria while the horizontal axis represents average occurrence of *A. flavus.*

**Figure 7:**
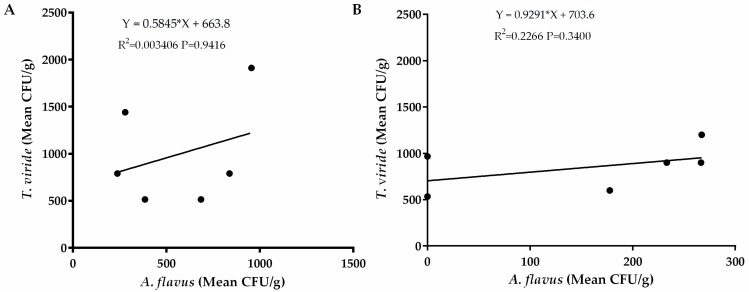
Regression analysis between occurrence of *A. flavus* and *Trichoderma viride* in soils sampled from (**A**) eastern and (**B**) western regions of Kenya. The vertical axis represents average occurrence (CFU/g) of *T. viride* while the horizontal axis represents average occurrence of *A. flavus*.

Regression analysis revealed a weak relationship between occurrence of *A. flavus* and *Pseudomonas* spp. in the western region (R^2^ = 0.03693) and the eastern region (R^2^ = 0.06126) as well as occurrence of *Bacillus* spp. in the western region (R^2^ = 0.196) and in the eastern region of Kenya (R^2^ = 0.03693). (Figure 6). There was also a weak relationship between occurrence of *Trichoderma viride* in both eastern (R^2^ = 003406) and western (R^2^ = 0.2266) regions of Kenya (Figure 7). These weak relationships led us into speculating that occurrence of the bacterial species had little influence on occurrence of *A. flavus* in the two regions. To ascertain whether these bacteria show potential for biocontrol, we carried out a preliminary assay for efficacy of both *Pseudomonas* spp. and *Bacillus* spp. against *A. flavus* growth in vitro. We found that none of the bacterial strains from both species had an inhibitory effect on *A. flavus* growth (data not shown).

### 2.6. Qualitative and Quantitative Determination of Aflatoxigenicity of A. Flavus Isolates 

**Figure 8:**
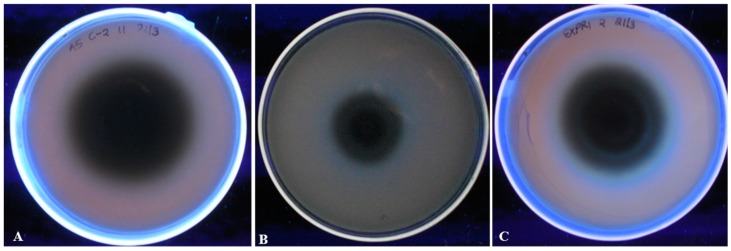
*A* screening assay of *A. flavus* isolate aflatoxigenicty using neutral red desiccated coconut agar. (**A**) A low aflatoxin producer isolate (**B**) A medium aflatoxin producer isolate (**C**) A high aflatoxin producer isolate. Presence of a blue fluorescence around the isolate indicates aflatoxin production.

Visual determination of aflatoxigenicity was done using Neutral red desiccated coconut agar (NRDCA) medium as described earlier by Atanda et al. [37]. For this study, we classified the isolates into three categories of high (scored with +++), mild (++) and low (+) aflatoxin producers. This was based on the intensity of a blue color around the fungal isolate upon observation under UV light (Figure 8). We noted a variation in the quantities of aflatoxins produced by *A. flavus* isolates across the study sites and the details of the aflatoxin levels is outlined in Table 2. Generally, isolates from the eastern region of Kenya produced higher levels of total aflatoxins as compared to those from the western region of Kenya. Particularly, 2 isolates from Makueni produced the highest total levels of aflatoxin at 144.75 ppb and 113.8 ppb and that from Kitui also resulted in a high toxin level (103.3 ppb) (Table 2). No aflatoxins were detected from 3 representative isolates from western and one from the eastern region (Table 2). The levels of aflatoxins were a reflection of the qualitative aflatoxin pre-screening assay using NRDCA method (Table 2) and in one case linked to a deletion of one of the genes screened using PCR.

## 3. Discussion

The eastern region of Kenya is a hot bed of aflatoxin contamination while the western region is one of the leading maize growing areas in the country that has shown low levels of aflatoxin contamination [11,39,40]. In this study, we compare the fungal and bacterial species in soil samples from western and eastern regions. Previous reports projected that a change in climatic conditions will have a marked impact on mycotoxin contamination of crops around the world [41]. This, however, needs to be confirmed using studies on distribution of mycotoxin-producing fungi under different ecologies and climatic conditions. In the current study, we have compared the occurrence and distribution of *A. flavus* in soils from two regions with contrasting agro-climatic conditions in Kenya and subsequently show their aflatoxin producing abilities. We further isolated bacteria from the soils from these regions while hypothesizing that they could be of biocontrol significance. 

From our findings, it was evident that significantly higher occurrence of *A. flavus* was recorded in soils from the eastern region compared to the western region. Generally, the eastern region of Kenya experiences hotter and drier climatic conditions compared to the western region. The eastern region is mainly classified as semi humid-semiarid while the western region is classified as sub-humid-semi-humid agro-ecological zone [34]. Prevailing environmental conditions of temperature, rainfall and humidity have been shown to affect the ability of fungi to infect, colonize and survive on crops as well as produce mycotoxins. Fluctuations in these parameters have been shown to influence quantities as well as community compositions of aflatoxin-producing fungi [17]. Furthermore, it has been demonstrated that *A. flavus* prefers climates with warmer tropical and sub-tropical conditions [42]. Payne et al. [32] reviews studies that have demonstrated how higher temperatures and drought increase *A. flavus* occurrence and aflatoxin production under field conditions. Our findings are consistent with earlier studies that reported a higher occurrence of *Aspergillus* spp. in drier areas of Makueni compared to humid regions [43]. Since the eastern region of Kenya experiences hotter and drier conditions compared to the western region, it is likely that this phenomenon played a key role in the observed profiles of *A. flavus* occurrence and distribution. These climatic patterns, (semi-arid with warm and dry conditions) experienced in the eastern region of Kenya are ideal for growth of aflatoxin-producing fungi and have further been implicated in influencing the density and distribution of *A. flavus* [22]. Lower occurrences of *A. flavus* as well as low levels of aflatoxins have further been reported in regions with adequate rainfall and lower temperatures [44]. 

The importance of cropping systems on occurrence, distribution and the ability of fungi to produce aflatoxins is well documented in mycotoxin-related studies [22,45,46]. *Aspergillus flavus* naturally inhabits the soil and decaying vegetation and has further been implicated in contamination of crops including maize, cotton, groundnuts, sorghum and millet [45,47]. Particularly, soil and plant debris act as reservoirs of fungal inoculum with reports implicating the debris in supporting survival and reproduction of *A. flavus*. This is because *A. flavus* is a saprophytic fungus that depends on organic matter for survival [16]. In the current study, all the *A. flavus* recorded was isolated from soils sampled from fields under maize cultivation. On the other hand, we did not record any *A. flavus* in soils sampled from fields under sugarcane cultivation. While we understand that fungal ecology varies depending on the species in question, we attribute the current finding to the fact that *A. flavus* is more likely to colonize certain crops as compared to others and as a result, a higher occurrence is likely to be found in soils under such cropping systems. This preference has previously been demonstrated by Bandyopathyay et al. [47], showing that maize was more contaminated by aflatoxins than sorghum and millet as a result of higher *A. flavus* colonization. Similar results were also reported in the India where less *A. flavus* colonization and subsequently less aflatoxin contamination of pearl millet compared to maize was reported [46]. In Africa, occurrence of *A. flavus* in maize-growing fields has been profiled and reported [48,49]. Colonization of sugarcane by aflatoxin-producing fungi as well as occurrence of these fungi in fields under sugarcane cropping systems has not been reported in the region. This, however, has been shown in other parts of the world [50,51]. We therefore propose that fungal ecologies are affected by the type of plant debris in the soil and this is likely to play a vital role in the density of fungi present.

Identification and characterization of aflatoxin-producing fungi requires a polyphasic approach to fully confirm their taxonomic status [9,10]. Morphological characterization of isolated fungi is achieved by analyzing fungal attributes such as colony color and conidia morphology while distinction of fungal groups is further achieved through chemical analysis for presence of mycotoxin production. Since species identification based on these two approaches may not be adequate, owing to the complexity of classes of fungi, a further test using molecular tools is vital. In aflatoxin-producing fungi, this has been achieved through studies targeting presence or absence of one or more genes in the aflatoxin biosynthetic pathway [52,53,54]. Relating results from such screening with the respective aflatoxin profiles helps to sufficiently characterize *Aspergillus* spp. In the current study, we screened fungal isolates for the presence of *aflD* and *aflQ* genes in DNA and isolated and detected *aflQ* in all the samples studied while all but two isolates did not show presence of *aflD.* The fungal cultures that lacked the *aflD* gene following PCR also showed low florescence intensities upon screening on NRDCA and ultimately lower levels of aflatoxins. The two genes have been previously used in analysis and characterization of *Aspergillus* spp. [23,55]. This study further corroborates the finding by Probst et al. [21] indicating that some *A. flavus* strains from Kenya had gene deletions that could result in low levels of aflatoxins.

We observed a positive relationship between occurrences of *A. flavus* and *Pseudomonas* spp. in eastern and western regions but a negative relationship between *A. flavus* and *Bacillus* spp. in western Kenya. Nevertheless, the R^2^ were too low for us to make any conclusions on whether these bacteria could be of any biocontrol significance. Although preliminary exclusive competitive bioactivity assay showed that the bacteria were not bioactive against *A. flavus*, previous studies have demonstrated the efficacy of various microorganisms including bacteria as fungal biocontrol agents [26,56,57,58]. It is possible that they may not have competitive exclusion ability against *A. flavus* and that another/other microbes could be responsible. Also, since there are many biological control mechanisms exhibited by microorganisms, it is not possible to overrule the potential of these microbes against *A. flavus* until conclusive tests are performed. Further, positive relationship analysis between *A. flavus* and *Trichoderma viride* isolates although not significant at *p* > 0.05, indicated that the isolates from these regions may not have the inhibitory capability needed to competitively exclude *A. flavus*. *Trichoderma* isolates from other regions have been successfully used in biological control of *A. flavus* [57,59].

## 4. Conclusions

In summary, our results demonstrated that eastern and western regions of Kenya harbor different quantities of *A. flavus*, *T. viridae*, *Pseudomonas* spp. and *Bacillus* spp. This could be due to varied factors like cropping patterns and environmental factors. This information, if combined with other forecasting tools like geographic information systems, can be part of the prediction tools for aflatoxin hot spots in Kenya and the east Africa region. It would also be important to investigate how the proposed prediction model would influence the dissemination and application rate of *A. flavus* biocontrol products currently at the commercialization stage in Kenya and several other African countries as the inoculum levels of aflatoxigenic *A. flavus* to be combated are varied across regions. In future we also recommend a metagenomics approach that has better resolution in understanding microbial diversity as well as circumvent limitation that come with culture-based methods.

## 5. Materials and Methods

### 5.1. Study Sites and Soil Sample Collection

Soil samples were collected from the eastern region (n = 80) from maize growing fields and western regions (n = 120 from maize growing fields and n = 80 sugarcane growing fields) of Kenya. The eastern region is in the arid and semiarid lands (ASALs) of Kenya with annual average temperature of 24 °C and annual average rainfall of 300–600 mm [60]. It is an aflatoxin endemic region and some of the worst cases of acute aflatoxicosis have been reported there [11]. The sampled sites in the eastern region included Makueni, Kitui, Machakos and Yatta. These areas have two maize planting seasons from March to May and October to December. On the other hand, the western region has average annual temperatures of 20.6 °C and average annual rainfall of 1971 mm [60]. Maize is grown from February to September (long rain season) and October to December (short rain season). The sampled sites in the western region were as follows for maize growing fields—Bukura, Mabanga, Eshitsitswi, Sikusa, Sang’alo and Mlimani at the Kenya Agricultural Livestock Research Organization (KALRO) station in Kakamega. We further sampled soil from sugarcane growing fields at Lunza, Bukura, Handid, Sikusa and Malava. No cases of aflatoxin outbreaks have been reported in these locations [61]. Soils from the eastern region of Kenya were collected from farms previously under maize cultivation while those from western were sampled from farms under maize as well as sugarcane cultivation. At every site, independent collections of five 40 mm diameter cores to a depth of 12 cm, at randomly selected points (∼490 g soil each), were taken. In order to reduce large-scale site heterogeneity while retaining microscale heterogeneity, each group of five cores were gently mixed yielding a composited sample representing each of the four field replicate locations that were later mixed to make a composite site sample. The composites site sample was further pulverized before being stored at 4 °C to await isolation of fungi and bacteria. The pH of these soils was measured as described [62] and their profiles are summarized in Table 3.

### 5.2. Isolation and Identification of Fungal Species 

Composite soil samples (n = 4) for the eastern region and those from the western region (n = 6 from maize and n = 5 from sugarcane fields) were first serially diluted before fungal isolation using a protocol earlier described by Reference [63]. Briefly, one gram of each sample was dissolved in 9 mL of autoclaved distilled water and serially diluted to 10^−3^. This was repeated 3 times (biological replicates) on the same samples. An aliquot of each dilution (500 µL) was plated by spreading on modified Rose Bengal agar (MRBA) medium amended with 30 mg/L chloramphenicol, plates were then sealed and incubated in darkness for 7 days at 28 °C. This was replicated 3 times (technical replicates) for two of the biological replicates and four times (technical replicates) for the third biological replicate. The 10 plates (from technical replicates) were checked for fungal growth followed by counting of the colonies using a colony counter and this information was used to enumerate the average number of colony forming units per gram (CFU/g) of soil. A total of 10 plates were plated for each soil sample per location and the experiment was replicated 3 times. Emerging colonies were point-inoculated on potato dextrose agar (PDA) and incubated at 28 °C. The growing fungal cultures were subsequently sub-cultured until pure colonies were obtained. Pure fungal colonies were identified to the species level using cultural and morphological characteristics described as follows; *Aspergillus* species [64], *Fusarium* species [65], *Penicillium* species [66] and *Trichoderma* species [67]. Cultures of the genus *Aspergillus* were then transferred to *Aspergillus-flavus-parasiticus* agar medium (AFPA) and incubated at 28 °C in the dark for 5 days for reverse color identification [68]. Isolates showing an intense yellow-orange color on the base of the medium were considered *A. flavus* and therefore selected for subsequent experiments.

### 5.3. Determination of Aflatoxin-Producing Ability and Quantification of Aflatoxins in Different Fungal Isolates

Aflatoxigenicity of *A. flavus* isolates was first qualitatively determined in vitro using neutral red desiccated coconut agar media (NRDCA) medium with visualization under UV light (at 340 nm) as described in Reference [37]. To determine the quantities of aflatoxins produced by fungal cultures, isolated *A. flavus*, one high and one low producer isolate per sample based on fluorescence experiment above were purposively selected and point-inoculated on aflatoxin-inducing Yeast Extract Sucrose (YES) agar medium according to Reference [69]. The isolates were incubated at 28 °C for 7 days in the dark. Aflatoxins were then extracted from 2 g of agar medium using the RIDASCREEN^®^ Aflatoxin Total (Art. No.: R4701; R-Biopharm AG Darmstadt, Germany) according to the manufacturer’s instructions. The kit is optimized to extract aflatoxin B_1_, B_2_, G_1_ and G_2_ that we herein refer to as total aflatoxins. Total aflatoxin extracts were then quantified by measuring absorbance of the samples and the controls (0 ppb (zero standard), 0.05 ppb, 0.15 ppb, 0.45 ppb, 1.35 ppb, 4.05 ppb aflatoxin B1 methanol/water, ready to use) on the same microtiter plate using a microtiter spectrophotometer at 450 nm. The measurement is made photometrically at 450 nm; the absorption is inversely proportional to the aflatoxin concentration in the sample. Actual total aflatoxin concentrations were calculated from RIDASCREEN^®^ enzyme immunoassays using a special software, the RIDA^®^SOFT Win (Art. No. Z9999; R-Biopharm AG Darmstadt, Germany). The lowest detection limit for the kit is 1.75 ppb. 

### 5.4. Molecular Characterization of A. flavus Cultures through Detection of aflD and aflQ Genes

Following aflatoxin quantification, one isolate was selected from each of the study sites for molecular analysis. Fungal genomic DNA was isolated from the isolates using a protocol described by Dehghan et al. [70]. Briefly, 7 day-old fungal mycelia growing on PDA were frozen in liquid nitrogen and ground to a fine powder using a mortar and pestle. The mycelial powder was then re-suspended in DNA extraction buffer containing 50 M Tris-HCl, (pH 8.0), 50 mM EDTA, 3% SDS, 1% *ß*-mercaptoethanol and 2 mg/mL Proteinase-K. The suspension was incubated at 65 °C for 30 min and the cellular debris removed by centrifugation at 7826× *g* for 15 min. After addition of 0.25 mg/mL RNase A, the suspension was incubated at 37 °C for 30 min, extracted once with phenol-chloroform-isoamyl alcohol (25:24:1) and once with chloroform-isoamyl alcohol (24:1). The DNA was precipitated by addition of an equal volume of isopropanol and 3M sodium acetate, followed by centrifugation at 7826× *g* for 30 min. The DNA pellet was washed using 70% ethanol, dried, re-suspended in nuclease-free water and stored at −20 °C until needed for PCR.

For PCR analysis, two genes involved in aflatoxin biosynthesis were targeted. Gene-specific primers for amplification of *aflD* (*aflD* F-5′ACC GCT ACG CCG GCA CTC TCG GCA C-3′ *aflD*-R-5′ GTT GGC CGC CAG CTT CGA CAC TCC G-3′) and *aflQ* (*aflQ*-F-5′TTA AGG CAG CGG AAT ACA AG-3′ *aflQ* R-5′ GAC GCC CAA AGC CGA ACA CAA A 3′) [69] were used in this study. PCR amplification was carried out in a 25 µL reaction mixture comprising 10 × PCR buffer, 1 unit/reaction Taq polymerase (Kapa Biosystems Inc., Wilmington, MA, USA), 0.2 µM of each primer and 1 ng/µL of template DNA. Amplification was done using a thermocycler (Eppendorf, Hamburg, Germany) with the following conditions; pre-heating at 94 °C for 5 min followed by 30 cycles of denaturation at 94 °C for 30 s, annealing at 50 °C for 30 s and extension at 72 °C for 1 min for both primers. A final 10-min extension step at 72 °C was also included. The PCR products were electrophoresed on 1% agarose gel in TAE buffer stained with 1 µL SYBR™ green. The products were visualized under UV light in a trans-illuminator after running the gel at 80 volts for 1 h. 

### 5.5. Isolation and Characterization of Recovered Bacteria

To isolate bacteria, 1 g of soil was first dissolved in 9 ml of autoclaved distilled water and serially diluted to 10^−3^ to reduce the number of emerging colonies for effective counting. A 500 µL aliquot of the dilution was then spread onto plates containing nutrient agar (NA) medium (Oxoid), plates sealed and incubated at 28 °C for 48 h. All emerging bacterial colonies were counted and used to enumerate CFU per gram of soil. A loopful of bacterial isolates were then individually picked from the master plates and streaked onto fresh NA plates to obtain pure colonies. We isolated several bacterial species but focused on *Bacillus* or *Pseudomonas* genera because these have been implicated in biocontrol activities against aflatoxin-producing fungi [71]. To identify these 2 bacterial species, biochemical tests including gram staining and oxidase activity on media were carried out as described by Yazdankhah et al. [72].

For molecular characterization of bacterial isolates, the 16S rRNA gene was amplified and sequenced. DNA was first extracted from 48 h-old pure bacterial colonies grown on NA medium using the DNeasy Ultraclean Microbial Kit (Qiagen, Hilden, Germany) according to the manufacturer’s instructions. The DNA (1 ng/µL) eluted in TE buffer was used as a template for PCR amplification with universal 16S rRNA primers (16S F-5′ AGA GTT TGA TCC TGG CTC AG 3′ and 16S R-5′ CGG TTA CCT TGT TAC GAC TT 3′) adopted from Jagoueix et al. [73] in a reaction mixture containing 10X PCR buffer, 1 unit/reaction Taq (Kapa Biosystems Inc., Wilmington, MA, USA) and 0.2 µM of each primer. PCR conditions were as follows; denaturation at 94 °C for 5 min followed by 30 cycles comprising of denaturation at 94 °C for 30 s, annealing at 50 °C for 30 s, extension at 72 °C for 1 min and a final extension of 72 °C for 10 min. PCR products were confirmed on a gel, purified using the QIAquick PCR purification kit (Qiagen) and sent for Sanger sequencing using the forward primer.

Sequences were retrieved from Biosciences East and Central Africa Hub, International Livestock Research Institute (BecA, ILRI) and trimmed to remove those of primers before using them to generate phylogenetic trees. The edited sequences were first deposited in the national center for biotechnology information (NCBI) with accession numbers KY379939, KY379940, KY379941, KY379942, KY379943, KY379944, KY379945, KY379946, KY379947, KY379948, KY379949, KY379950 and then used to query the basic local alignment search tool (BLAST) algorithm at NCBI for related sequences. These sequences were retrieved and then aligned using the Clustal algorithm in Molecular Evolutionary Genetics Analysis (MEGA) 7.0 software with default settings. The alignment was used to construct a phylogenetic tree using the Maximum Likelihood method in MEGA [38]. A pairwise deletion mode with Poisson correction and a bootstrap of 1000 replicates was also included. The trees were rooted using a 16S rDNA sequence from *Xanthomonas floridensis*.

### 5.6. Data Analysis 

A generalized linear model (GLM) was employed to perform analysis of variance (ANOVA) on all data sets. Tukey’s HSD test was used to compare means and determine significant differences among data sets at 95% confidence interval in SAS version 9.1.3 (SAS Institute Inc., Cary, NC, USA). Comparisons between *A. flavus* and *Trichoderma viride* as well as the bacterial isolates across study regions (western and eastern) were done using a non-parametric *t* test at *p* ≤ 0.05. Regression analysis were performed using Graph Pad Prism version 6 (San Diego, CA, USA).

## Figures and Tables

**Table 1:** Comparison of fungal and bacterial occurrence across western and eastern regions using *t* test.

Identity	Region	Mean	SD	*t*	df	*p*-Value
	W	157.5	51.56			
*A. flavus*	E	689.8	147.3	3.998	8	0.004 *
	W	211.1	72.3			
*A. parasiticus*	E	202.7	65.47	0.0802	8	0.938
	W	844.44	99.51			
*Trichoderma viride*	E	1116	315.7	1.146	8	0.285
	W	3.837 × 10^4^	1.029			
*Bacillus* spp.	E	1.243 × 10^4^	0.8230	1.799	8	0.1097
	W	1.112 × 10^4^	0.4166			
*Pseudomonas* spp.	E	0.8188 × 10^4^	0.5838	0.4215	8	0.6845

W-Western region, E-Eastern region, SD-standard deviation. df-degrees of freedom, * denotes significance (*p* ≤ 0.05).

**Table 2:** Fluorescence intensity under Ultra Violet light and aflatoxin levels of *A. flavus* isolates.

Isolate No.	Source of Isolate Where Isolated	Fluorescence Intensity on NRDCA	Total Aflatoxins (ppb)
1	KALRO	+	ND
2	Mabanga	+	ND
3	Sikusa	++	3.8
4	Sang’alo	+	ND
5	Kitui	+++	103.3
6	Kitui	+++	78.8
7	Yatta	+	ND
8	Makueni	++	2.9
9	Makueni	+++	144.75
10	Makueni	+++	113.8
11	Machakos	+++	42.9
12	Machakos	++	2.9

Isolates labeled 1–4 were from the western region while 5–12 were from the eastern region. NRDCA-Neutral red desiccated coconut agar. ppb- parts per billion. +++ -High, ++ -Mild, + -Low aflatoxin producers. Total aflatoxin levels represent a total of AFB1, AFB2, AFG1 and AFG2 as extracted and quantified using the Ridascreen kit.

**Table 3:** Profiles of soil pH from sampled areas.

Sample Ref.	Soil pH	Class
**Eastern province**		
Makueni	7.28	Slightly alkaline
Kitui	5.84	Medium acidic
Machakos	5.59	Medium acidic
Yatta	5.45	Medium acidic
**Western province**		
Bukura	5.37	Medium acidic
KALRO	5.27	Medium acidic
Mabanga	4.82	Strongly acidic
Eshitsitswi	5.04	Medium acidic
Sangalo	4.95	Strongly acidic
Sikusa	5.49	Medium acidic
